# IL-21 signaling promotes the establishment of KSHV infection in human tonsil lymphocytes by increasing differentiation and targeting of plasma cells

**DOI:** 10.3389/fimmu.2022.1010274

**Published:** 2022-12-07

**Authors:** Nedaa Alomari, Jennifer Totonchy

**Affiliations:** Biomedical and Pharmaceutical Sciences, Chapman University, Irvine, CA, United States

**Keywords:** Gamma herpesvirus, KSHV, HHV-8, B lymphocyte, KSHV transmission, IL-21

## Abstract

**Introduction:**

Factors influencing Kaposi’s sarcoma-associated herpesvirus (KSHV) transmission and the early stages of KSHV infection in the human immune system remain poorly characterized. KSHV is known to extensively manipulate the host immune system and the cytokine milieu, and cytokines are known to influence the progression of KSHV-associated diseases. Our previous work identified the early targeting of plasma cells for KSHV infection. In this study, we examine whether IL-21, a cytokine known to profoundly influence plasma cell fate, influences the early stages of KSHV infection in B lymphocytes.

**Methods:**

Using our unique model of ex vivo KSHV infection in tonsil lymphocytes, we investigate the influence of IL-21 supplementation, IL-21 neutralization, the distribution of IL-21 receptor on B cell subsets and IL-21 secreting T cell subsets on the establishment of KSHV infection in human B cells.

**Results:**

We show that IL-21 signaling promotes KSHV infection by promoting both total plasma cell numbers and increasing KSHV infection in plasma cells as early as 3 days post-infection. We further demonstrate that the synergistic effect of KSHV infection and IL-21 treatment on plasma cell frequencies is due to differentiation of new plasma cells from naïve B cell precursors. We examine T cells secreting IL-21 in our tonsil specimens and determine that IL-21 producing CD8+ central memory T cells are correlated with plasma cell frequencies and KSHV targeting of plasma cells.

**Discussion:**

These results demonstrate the novel finding that differentiation of new plasma cells is involved in the early stages of KSHV infection in B cells, and that IL-21 signaling can potentiate this effect thereby increasing the overall magnitude of KSHV infection at early timepoints. These results suggest that IL-21 signaling represents a host-level susceptibility factor for the establishment of KSHV infection.

## 1 Introduction

Kaposi’s Sarcoma Herpesvirus (KSHV) is a lymphotropic gamma-herpesvirus, originally discovered as the causative agent of Kaposi Sarcoma (KS) (1). KS is a highly proliferative tumor derived from lymphatic endothelial cells (2). KSHV is also associated with the B cell lymphoproliferative diseases, Primary Effusion Lymphoma (PEL) and Multicentric Castleman’s Disease (MCD) (3, 4), as well as the inflammatory disorder KSHV inflammatory cytokine syndrome (KICS) (5). KSHV is linked to 1% of all human tumors, and the World Health Organization (WHO) has classified it as class I carcinogen (6, 7). KSHV infection is asymptomatic in most healthy individuals, and KSHV-associated malignancies arise primarily in immunocompromised patients. Indeed, KS remains one of the most common cancers in people living with HIV/AIDS (8).

The geographical distribution of KSHV is not ubiquitous. KSHV infection is endemic in sub-Saharan Africa and in the Mediterranean basin. KSHV prevalence is also high in subpopulations in other parts of the world such as men who have sex with men (MSM). Saliva is the only secretion where KSHV DNA is commonly detected (9), and, based on this, person-to-person transmission of KSHV is thought to occur *via* saliva. The oral lymphoid tissues are rich in KSHV target cell types, including lymphatic endothelial cells and B cells, and are therefore a likely site for the initial establishment of KSHV infection in a new human host. However, the exact mechanisms for KSHV transmission and how environmental, behavioral and host factors influence transmission and early infection events remain to be definitively established. This gap in our understanding affects our ability to find efficient strategies to decrease the transmission or influence host-level susceptibility to KSHV infection. We previously analyzed susceptibility of B cells to KSHV infection in a cohort of human tonsil samples with diverse race, sex and age distributions. In this study, between 0.7% and 4% of tonsil B cells were GFP+ at 3 dpi and donor-to-donor variability of infection rate could not be linked to demographic factors. Moreover, this study demonstrated that CD138+ plasma cells are rare in tonsil but are preferentially targeted early in KSHV infection (10). Our ongoing research seeks to identify, and mechanistically characterize, factors that influence variable susceptibility to KSHV infection in the tonsil as a model for identifying potentially modifiable host susceptibility factors. It is important to note that, in this context, it is the highly susceptible and highly refractory “outlier” specimens that may ultimately prove the most informative in identifying these critical susceptibility factors.

Cytokine dysregulation is strongly linked to the pathogenesis of KSHV-associated lymphoproliferations (11). However, their contribution to the early stages of KSHV infection and whether the cytokine milieu in the oral lymphoid tissues contributes to host-level susceptibility to KSHV infection is unclear. IL-21 is a pleiotropic cytokine that has diverse effects on B cell, T cell, macrophage, monocyte, and dendritic cell biology. It is produced mainly by natural killer T (NKT) cells and CD4+ T cells, including follicular helper (Tfh) cells (12). The IL-21 receptor is expressed by several immune cells, including B and T cells and is comprised of a unique IL-21R subunit and the common cytokine receptor *γ* chain (CD132), which is also part of the receptor for IL-2, IL-4, IL-7, IL-9, and IL-15 (13). IL-21 plays a critical role in B cell activation and expansion (14), as well as B cell differentiation to immunoglobulin (Ig)-secreting plasma cells. The regulation of maturation of B cells into plasma cells is driven by the several transcription factors including Blimp1 and Bcl6 (15), which can both be induced by IL-21 signaling, indicating that IL-21 is an important regulator of plasma cell differentiation (16, 17). IL-21 has been studied in the pathogenesis of chronic lymphocytic choriomeningitis virus (LCMV) infection, influenza virus, and, most relevant to this study, IL-21 plays an important role in the early establishment of murine gammaherpesvirus 68 (MHV68) infection in mice (18–20). Moreover, IL-21 induces differentiation of B-lymphoblastoid cell lines into late plasmablast/early plasma cell phenotypes, and regulates the expression of many latent proteins in EBV^+^ Burkitt lymphoma cell lines (21, 22). Although IL-21 is canonically thought to act primarily in germinal centers, it is detected in interfollicular areas in MCD patients (23). There are no studies, to date, examining the contribution of IL-21 to KSHV infection and KSHV-associated disease.

In this study, we test the hypothesis that IL-21 signaling plays a role in the early establishment of KSHV infection in plasma cells. We use our well-established model of *ex vivo* infection in human tonsil lymphocytes to test this hypothesis. As with our previous studies exploring factors influencing susceptibility of tonsil samples to KSHV infection, we concentrated data collection on 3 dpi as it is the earliest timepoint in which infection can be reliably detected. This strategy allows us to maximize our ability to identify sample-intrinsic susceptibility factors and minimize the contribution of artifacts that may accumulate over time in *ex vivo* cell culture. We show that supplementation of tonsil lymphocyte cultures with IL-21 enhances total KSHV infection. In the presence of IL-21, we observe increased levels of KSHV-infected plasma cells, and a synergistic increase in total plasma cell numbers in IL-21 treated, KSHV infected cultures. Interestingly, both plasma cell targeting and increased total plasma cells are significantly correlated with increased overall KSHV infection, indicating that plasma cell frequencies and/or targeting influences the establishment of infection in non-plasma cell subsets. We further explore the immunological mechanisms of this IL-21 effect by establishing which B cell types are responding IL-21, and what T cell subsets are producing IL-21 in our model. Finally, we show that the synergistic effect of IL-21 and KSHV on plasma cell frequencies is a result of B cell differentiation and proliferation.

These results identify IL-21 signaling as a factor that influences the establishment of KSHV infection in B lymphocytes *via* manipulation of plasma cell differentiation. Together with our previous work, this study underscores the importance of plasma cell biology in the initial establishment of KSHV infection in the oral lymphoid tissues and provides the first direct evidence that B cell differentiation influences the early stages of infection for KSHV.

## 2 Materials and methods

### 2.1 Reagents and cell lines

CDw32 L cells (CRL-10680) were obtained from ATCC and were cultured in DMEM supplemented with 20% FBS (Sigma Aldrich) and Penicililin/Streptomycin/L-glutamine (PSG/Corning). For preparation of feeder cells CDw32 L cells were trypsinized and resuspended in 15 ml of media in a petri dish and irradiated with 45 Gy of X-ray radiation using a Rad-Source (RS200) irradiator. Irradiated cells were then counted and cyropreserved until needed for experiments. Cell-free KSHV.219 virus derived from iSLK cells [39] was a gift from Javier G. Ogembo (City of Hope). Human tonsil specimens were obtained from the National Disease Research Interchange (NDRI; www.ndriresource.org) or Cooperative Human Tissue Network (CHTN; www.chtn.org). Human fibroblasts for viral titering were derived from primary human tonsil tissue and immortalized using HPV E6/E7 lentivirus derived from PA317 LXSN 16E6E7 cells (ATCC CRL-2203). Antibodies for flow cytometry were from BD Biosciences and Biolegend and are detailed below. Recombinant human IL-21 was from Preprotech (200-21) and IL-21 neutralizing antibody was from R&D Systems (991-R2).

### 2.2 Isolation of primary lymphocytes from human tonsils

De-identified human tonsil specimens were obtained after routine tonsillectomy and shipped overnight on wet ice in DMEM+PSG. All specimens were received in the laboratory less than 24 hours post-surgery and were kept at 4˚C throughout the collection and transportation process. Lymphocytes were extracted by dissection and maceration of the tissue in RPMI media. Lymphocyte-containing media was passed through a 40µm filter and pelleted at 1500rpm for 5 minutes. RBC were lysed for 5 minutes in sterile RBC lysing solution (0.15M ammonium chloride, 10mM potassium bicarbonate, 0.1M EDTA). After dilution to 50ml with PBS, lymphocytes were counted, and pelleted. Aliquots of 5(10)^7^ to 1(10)^8^ cells were resuspended in 1ml of freezing media containing 90% FBS and 10% DMSO and cryopreserved until needed for experiments.

### 2.3 Infection of primary lymphocytes with KSHV

Lymphocytes were thawed rapidly at 37˚C, diluted dropwise to 5ml with RPMI and pelleted. Pellets were resuspended in 1ml RPMI+20%FBS+100µg/ml DNaseI+ 100µg/ml Primocin (In vivogen) and allowed to recover in a low-binding 24 well plate for 2 hours at 37˚C, 5% CO2. After recovery, total lymphocytes were counted and naïve B cells were isolated using Mojosort naïve B cell isolation beads (Biolegend 480068) or Naïve B cell Isolation Kit II (Miltenyi 130-091-150) according to manufacturer instructions. Bound cells (non-naïve B and other lymphocytes) were retained and kept at 37˚C in RPMI+20% FBS+ 100µg/ml Primocin during the initial infection process. 1(10)^6^ Isolated naïve B cells were infected with iSLK-derived KSHV.219 (per cell dose equivalent to the ID20 at 3 dpi on human fibroblasts) or Mock infected in a total of 400ul serum free RPMI in 12x75mm round bottom tubes *via* spinoculation at 1000rpm for 30 minutes at 4˚C followed by incubation at 37˚C for an additional 30 minutes. Following infection, cells were plated on irradiated CDW32 feeder cells in a 48 well plate, reserved bound cell fractions were added back to the infected cell cultures, and FBS and Primocin were added to final concentrations of 20% and 100µg/ml, respectively and recombinant cytokines or neutralizing antibodies were also added at this stage, depending upon the specific experiment. Cultures were incubated at 37˚C, 5% CO_2_ for the duration of the experiment. At 3 days post-infection, cells were harvested for analysis by flow cytometry and supernatants were harvested, clarified by centrifugation for 15 minutes at 15,000 rpm to remove cellular debris, and stored at -80˚C for analysis.

### 2.4 Flow cytometry analysis of lymphocyte subsets and KSHV infection

Approximately 5(10)^5^ lymphocytes per condition were harvested into a 96- well round bottom plate at day 0 (baseline) or at 3 days post-infection at 1500 rpm for 5 minutes. Cells were resuspended in 100μl PBS containing zombie violet fixable viability stain (Biolegend Cat# 423113) and incubated on ice for 15 minutes. Cells were pelleted at 1500rpm 5 minutes and resuspended in 200ul FACS Block (PBS + 2%FBS + 0.5%BSA) for 10 minutes on ice. Cells were pelleted at 1500rpm for 5 minutes and resuspended in 50μl of PBS with 0.5% BSA and 0.1% Sodium Azide (FACS Wash), containing antibody cocktails (see below) and incubated on ice for 15 minutes. After incubation, 150μl FACS Wash was added. Cells were pelleted at 1500rpm for 5 minutes followed by two washes with FACS Wash. Cells were collected in 200μl FACS Wash for flow cytometry analysis. Cells were analyzed using an LSR Fortessa X-20 cell analyzer (BD Biosciences). BD CompBeads (51-90-9001229) were used to calculate compensation for all antibody stains and methanol-fixed Namalwa cells (ATCC CRL1432) +/- KSHV were used to calculate compensation for GFP and the fixable viability stain. Flow cytometry data was analyzed using FlowJo software and exported for quantitative analysis in R as described below.

#### 2.4.1 Reagents for B cell frequencies

10μl BD Brilliant Stain Buffer Plus and antibodies as follows: IgD-BUV395 (2.5μl/test BD 563823), CD77-BV510 (2.0 μl/test BD 563630), CD138- BV650 (2μl/test BD 555462), CD27-BV750 (2μ/test BD 563328), CD19-PerCPCy5.5 (2.0μl/test BD 561295), CD38-APC (10μl/test BD 560158), CD20-APCH7 (2ul/test Biolegend 302313), IgM (2μl/test Biolegend 314524), IgG (2μl/test BD 561298), IgE (2μl/test BD 744319) and IL-21 receptor (2μl/test BD 330114).

#### 2.4.2 For baseline T cell frequencies

For baseline T cell frequencies 0.5(10)^6^ cells from baseline uninfected total lymphocyte samples were stained and analyzed as above with phenotype antibody panel as follows: CD95-APC (2μl, Biolegend 305611), CCR7-PE (2μl, BD 566742), CD28-PE Cy7 (2μl, Biolegend 302925), CD45RO-FITC (3μl, Biolegend 304204), CD45RA-PerCP Cy5.5 (2μl, Biolegend 304121), CD4-APC H7 (2μl, BD 560158), CD19-V510 (3μl, BD 562953), CD8-V450 (2.5μl, BD 561426).

### 2.5 Tracking dye and BrdU incorporation assays

Naïve and Plasma cells were labeled using a (cellTrace™ Violet BMQC Dye, Thermo Fisher Scientific) according to the manufacturer’s instructions. In brief, Cells were isolated and resuspended at 10^6^ cells/mL in working dye solution (1μM in 1× PBS) for 30 minutes at 37°C. Cells were washed with culture media and followed by infection. Bromodeoxyuridine (BrdU) was added to the infected cell cultures to final concentrations of 1mg/ml.

### 2.6 ICCS for IL-21 secretion

At 3dpi, cultures were treated for 6 hours with Protein Transport Inhibitor (Containing Monensin) (BD GolgiStop Catalog# 554724) according to manufacturer’s protocol to block cytokine secretion. Following incubation, approximately 1 million cells were harvested and viability and surface staining for T cell lineage markers was performed as described above. After the final wash, cells were fixed for 10 minutes in BD cytofix/cytoperm (51-2090KZ), pelleted and further treated for 10 minutes with cytofix/cytoperm+10% DMSO (super perm) to more effectively get intracellular antibodies into the nucleus. Intracellular antibodies, as follows, were diluted in 1x BD Permwash (51-2091KZ) and left on fixed cells overnight at 4˚C. RoR-γT-Alexa 647 (BD 563620, 5µl/test), FoxP3-BB700 (BD 566527, 5 µl/test), IL-21-BV421 (BD 564755, 5 µl/test), BLC6-BV711 (BD 561080, 5µl/test). Cells were then washed twice with 1x permwash and analyzed as described above.

### 2.7 RT-PCR

At 3 days post infection, 1(10)^6^ lymphocytes were harvested into an equal volume of Trizol and DNA/RNA shield (Zymo Research R110-250). Total RNA was extracted using using Zymo Directzol Microprep (Zymo Research R2060) according to manufacturer instructions. RNA was eluted in 10μl H2O containing 2U RNase inhibitors and a second DNase step was performed for 30 minutes using the Turbo DNA-Free kit (Invitrogen AM1907M) according to manufacturer instructions. One-step RT-PCR cDNA synthesis and preamplification of GAPDH, LANA and K8.1 transcripts was performed on 15ng of total RNA using the Superscript III One-step RT-PCR kit (ThermoFisher 12574026).

Duplicate no RT (NRT) control reactions were assembled for each sample containing only Platinum Taq DNA polymerase (Thermofisher 15966005) instead of the Superscript III RT/Taq DNA polymerase mix. After cDNA synthesis and 20 cycles of target pre-amplification, 2μl of pre-amplified cDNA or NRT control reaction was used as template for multiplexed real-time PCR reactions using TaqProbe 5x qPCR MasterMix -Multiplex (ABM MasterMix-5PM), 5% DMSO, primers at 900nM and probes at 250nM against target genes. All primer and probe sequences used in these assays have been previously published (10). Real time PCR was performed using a 40-cycle program on a Biorad real time thermocycler. Data is represented as quantitation cycle (Cq) and assays in which there was no detectable Cq value were set numerically as Cq = 41 for analysis and data visualization. The expression of each gene was normalized to that of a housekeeping gene *GAPDH*.

### 2.8 Statistical analysis

The indicated data sets and statistical analysis were performed in Rstudio software using ggplot2 (24), ggcorrplot (25), ggally (26) and tidyverse (27) packages. Statistical analysis was performed using rstatix (28) package. Specific methods of statistical analysis including Anova, independent t-test and Pearson correlations and resulting values for significance and correlation are detailed in the results text and/or corresponding figure legends. In all cases, we utilized repeated measures method for ANOVA because each unique tonsil specimen was used for all experimental conditions in the dataset.

## 3 Results

### 3.1 IL-21 supplementation increases KSHV infection in tonsil B lymphocytes

**Figure 1:**
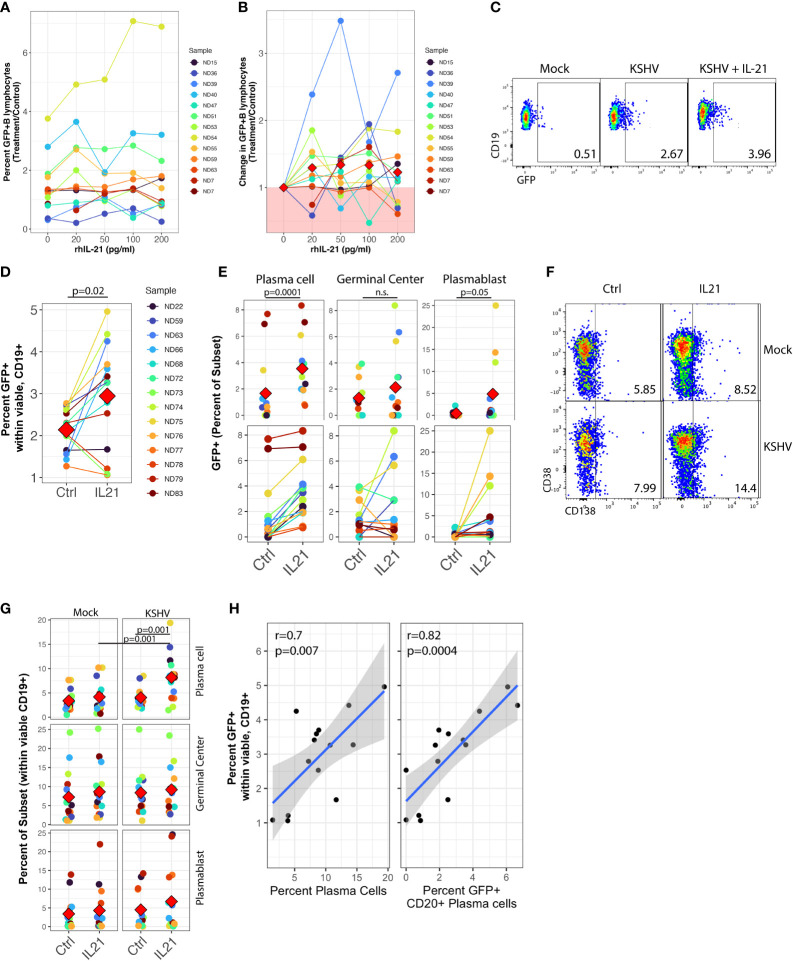
IL-21 supplementation increases overall KSHV infection and plasma cell frequencies. Naïve B Lymphocytes from 12 tonsil donors were infected with KSHV.219 and cultured with indicated doses of recombinant human IL-21 and analyzed at 3dpi by flow cytometry **(A)** the dose effect of IL-21 supplementation on viable/CD19+/GFP+ B lymphocytes. **(B)** data as in **(A)** normalized to the untreated control for each specimen. Colored points denote unique tonsil specimens and can be compared between **(A-C)** Representative flow cytometry showing viable, CD19+ B lymphocytes expressing GFP as an indicator of KSHV infection in Mock, KSHV (untreated) and KSHV+IL-21 (100pg/ml treated) **(D)** summary data for 12 additional tonsil donors analyzed as in **(A)** with 100pg/ml IL-21 treatment. Red diamonds indicate group means. p=0.02, F=6.4 comparing untreated and IL-21 treated via one-way repeated measures ANOVA. Tonsil lymphocyte specimens from **(D)** were stained for B cell immunophenotypes and analyzed by flow cytometry and GFP for KSHV infection to determine **(E)** GFP frequencies within B cell subsets for KSHV-infected cultures and **(G)** total B cell subset frequencies within viable/CD19+ for each condition. Top panels in **(E, G)** show individual sample quantities and means (red diamonds) for the indicated subsets and bottom panel in **(E)** shows trends of increased/decreased subset targeting on a per-sample basis. Colored points denote unique tonsil specimens and can be compared between **(D–G)** Representative flow cytometry data showing increased plasma cell frequencies with IL-21 treatment and KSHV infection. **(H)** Pearson correlation between overall GFP+ B cells in KSHV-infected, IL-21 treated cultures as in **(D)** and the frequency of plasma cells (left) and infection of plasma cells (right). Blue line is linear model regression and grey shading indicates 95% confidence interval. Count-level data can be found in **Supplementary Table 1** to provide an idea of event numbers used to generate these percentage values. Note that these are not absolute cell numbers because counting beads were not used in the analysis.

In order to examine the impact of IL-21 on the establishment of KSHV infection, we performed Mock infection or KSHV infection in 13 unique tonsil samples and left the cultures untreated or supplemented the resulting cultures with varying concentrations of recombinant human IL-21(IL-21). Doses of IL-21 used for these experiments were based upon immunoassay results showing that IL-21 is present in tonsil lymphocyte cultures with an average concentration of 20pg/ml at 3dpi in both mock and KSHV-infected cultures (**Supplementary Figure 3A**). At 3 dpi, we analyzed these cultures for GFP+ B lymphocytes by flow cytometry to assess the magnitude of KSHV infection (**Figures 1A, B**). As expected, the specimens had high variability in their baseline susceptibility. However, we observed a trend of increased infection in response to IL-21 treatment, and the effect seems to be stronger in the more susceptible samples (**Figure 1A**). Normalization of the data to each specimen’s untreated control reveals that 10/13 tonsil samples show some level of increased infection upon treatment with 100pg/ml of IL-21.

We then repeated these supplementation experiments with only the 100pg/ml dose of recombinant IL-21 in an additional 12 tonsil specimens and examined both overall infection and subset-specific responses in these cultures at 3 dpi using our established B cell immunophenotyping panel (**Table 1** and **Supplementary Figure 1A**) (10). As in the initial dataset, this analysis shows increased GFP+ B lymphocytes in response to recombinant IL-21 (**Figure 1C**) in most tonsil specimens examined (**Figure 1D**), and the difference in GFP+ B lymphocytes with IL-21 treatment compared to control was statistically significant in this cohort of specimens *via* one-way repeated measures ANOVA(p=0.02, F=6.4).This increase in infection was not associated with alterations in the frequency of viable B cells in the cultures (**Supplementary Table 1**).

**Table 1:** Lineage definitions for lymphocyte subsets used in the study.

B Lymphocytes	
Subset	Molecular Markers
Plasma	CD19**^+^,** CD20**^+/-^**, CD138**^+(Mid to High)^**, CD38**^-^**
Transitional	CD19**^+^,** CD138**^-^**, CD38**^Mid^**, IgD**^+ (Mid to High)^**
Plasmablast	CD19**^+^,** CD138**^-^**, CD38**^High^**, IgD**^+/- (mostly -)^**
Germinal Center	CD19**^+^,** CD138**^-^**, CD38**^Mid^**, IgD**^-^**
Naïve	CD19**^+^,** CD138**^-^**, CD38**^Low^**, CD27**^-,^** IgD**^+ (Mid to High)^**
Marginal Zone Like (MZ-Like)	CD19**^+^,** CD138**^-^**, CD38**^Low^**, CD27**^+ (Mid to High)^**, IgD**^+ (Mid to High)^**
Memory	CD19**^+^,** CD138**^-^**, CD38**^Low^**, CD27**^+ (Mid to High)^**, IgD**^-^**
Double Negative	CD19**^+^,** CD138**^-^**, CD38**^Low^**, CD27**^-^**, IgD**^-^**
**T lymphocytes**	
**Subset**	**Molecular Markers**
CD4+	CD19**^-^**, CD4**^+(Mid to High)^,** CD8**^-^**
CD8+	CD19**^-^**, CD4**^-^**, CD8**^+ (Mid to High)^**
Naïve	CD19**^-^**, CD4+ or CD8+, CCR7**^+(High)^**, CD45RA**^+(Mid to High)^**, CD45RO**^-^**, CD28**^+^**, CD95**^-^**
Stem Cell Memory	CD19**^-^**, CD4+ or CD8+, CCR7**^+(High)^**, CD45RA**^+(Mid to High)^**, CD45RO**^-^**, CD28**^+^**, CD95**^+ (Low to Mid)^**
Central Memory	CD19**^-^**, CD4+ or CD8+, CCR7**^+^**, CD45RA**^-^** CD45RO**^+(Mid to High)^**, CD28**^+ (Mid to High)^**
Transitional Memory	CD19**^-^**, CD4+ or CD8+, CCR7**^-^**, CD45RA**^-^** CD45RO**^+(Mid)^**, CD28**^+ (Mid to High)^**
Effector Memory	CD19**^-^**, CD4+ or CD8+, CCR7**^-^**, CD45RA**^-^** CD45RO**^+(Mid)^**, CD28**^-^**
Terminal Effector Memory	CD19**^-^**, CD4+ or CD8+, CCR7**^-^**, CD45RA**^-^** CD45RO**^-^**, CD28**^-^**
TEMRA CD4+ Cells	CD19**^-^**, CD4+ or CD8+, CCR7**^-^**, CD45RA**^+(High)^,** CD45RO**^-^**, CD28**^-^**
Tfh	CD19-, CD4+, CD8-, PD-1+, CXCR5+, CD127+, Intracellular BCL-6+/-
Treg	CD19-, CD4+, CD8-, CD25+, CD127+, Intracellular FoxP3+
Th17	CD19-, CD4+, CD8-, Intracellular ROR gamma T+

### 3.2 IL-21 increases both plasma cell frequency and targeting

To examine whether IL-21 treatment altered the B cell subset-specific distribution of KSHV infection in these experiments, we quantitated the percent of infected (GFP+) cells that corresponded to each B cell immunophenotype to determine the between-subsets distribution of KSHV infection in control or IL-21 treated cultures. One-way repeated measures ANOVA analysis indicates that IL-21 supplementation significantly increased KSHV infection within both plasma cells (p=0.0001, F=29.5) and plasmablasts (p=0.05, F=4.6). We also observed a notable but non-significant increase in infection of germinal center cells (**Figure 1E**, top) In this analysis, the effect of IL-21 on infection within plasma cells was very consistent with increased targeting observed in nearly every sample, while in GC and plasmablasts there was a clear demarcation of samples that were either responsive or non-responsive to IL-21 supplementation (**Figure 1E**, bottom).

We next examined whether IL-21 supplementation was associated with alterations in overall B cell frequencies in either Mock or KSHV-infected cultures. Two-way repeated measures ANOVA analysis revealed a highly significant increase in total plasma cell frequency (**Figure 1F**) associated with both IL-21 treatment (p=0.002, F=14.1) and KSHV infection (p=0.002, F=14.4) with a significant interaction of the two variables (p=0.001, F=17.8), indicating that KSHV and IL-21 synergistically increase total plasma cell numbers in our *ex vivo* cultures (**Figure 1G**). Neither infection nor treatment had a significant effect on germinal center cell frequencies, but there was a significant main effect of KSHV infection on frequencies of plasmablasts in these cultures (p=0.03, F=6). *Post hoc* paired T tests revealed significant differences with IL-21 treatment on total plasma cells (p=0.001) for the KSHV-infected conditions only, and in IL-21 treated conditions there was a significant difference between Mock and KSHV cultures for total plasma cells (p=0.001). To determine whether the increase in total plasma cell frequency and/or increased infection of plasma cells was directly correlated with the effect of IL-21 on total KSHV infection, we performed linear model regressions and analyzed the results using Pearson’s method (**Figure 1H**). These results reveal significant linear correlations between total GFP+ B cells and total plasma cell frequency (r=0.7, p=0.007) as well as the frequency of infection within CD20+ plasma cells (r=0.82, p=0.0004). One potential mechanism for increased GFP frequencies in IL-21 treated cultures is IL-21-mediated induction of lytic replication resulting in spread of the virus to additional B cells. To examine this possibility, we performed RT-PCR for LANA (latent) or K8.1 (lytic) gene expression in KSHV-infected control or IL-21 supplemented cultures. These results show no significant change in expression of either LANA or K8.1 in IL-21 treated cultures, indicating that IL-21 is not globally inducing lytic replication in treated cultures (**Supplementary Figure 3B**). Taken together, this data shows that (1) IL-21 treatment promotes the establishment of KSHV infection in human tonsil B lymphocytes (2) KSHV and IL-21 synergistically promote increased plasma cell frequencies at 3 dpi and (3) both plasma cell frequencies increased plasma cell infection at 3 dpi significantly correlate with the IL-21-mediated increase in total KSHV infection.

### 3.3 Neutralization of IL-21 does not inhibit KSHV infection

**Figure 2:**
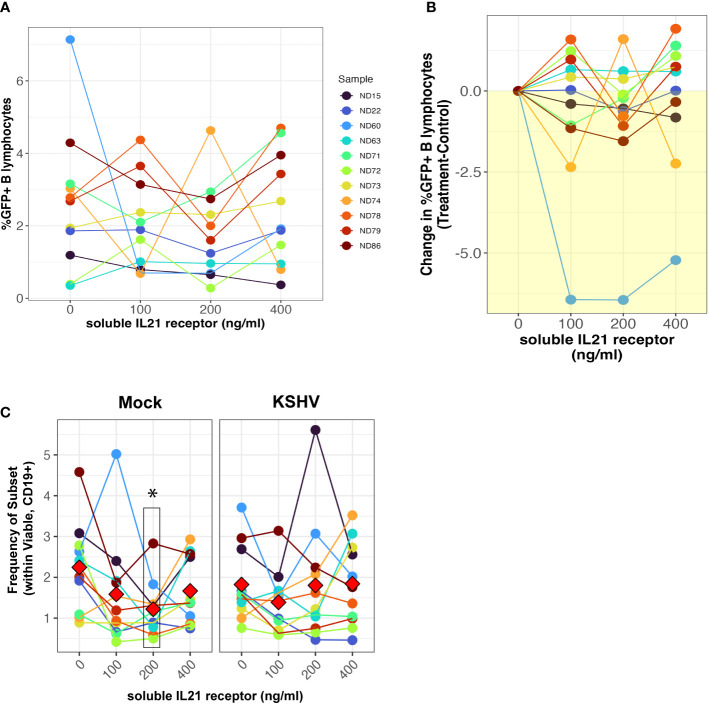
IL-21 neutralization does not affect the establishment of KSHV infection. Naïve B cells from 11 unique tonsil specimens were Mock or KSHV-infected and indicated concentrations of soluble IL-21 receptor was added to the resulting total lymphocyte cultures. Cultures were analyzed at 3 dpi for B lymphocyte immunophenotypes and the distribution of KSHV infection *via* GFP expression. Total GFP+ within viable/CD19+ B lymphocytes represented as **(A)** raw percentages or **(B)** normalized to the untreated control for each tonsil sample. **(C)** Frequencies of plasma cells (CD138+ within viable/CD19+) in the cultures. Colors denote unique tonsil specimens and can be compared between the panels. Red diamonds indicate group means. Paired T-tests showed significant effect of 200ng/ml soluble IL-21 receptor on total plasma cells (p=0.02) in Mock cultures only.

We next wanted to determine whether neutralization of the natively-secreted IL-21 in our tonsil lymphocyte cultures would affect the establishment of KSHV infection. To do this, we performed infections with Mock or KSHV-infection in 11 unique tonsil specimens, included varying concentrations of a soluble recombinant IL-21 receptor in the resulting cultures, and assessed the magnitude and distribution of KSHV infection at 3 dpi by flow cytometry. There was no inhibition of KSHV infection in B cells when IL-21 is neutralized (**Figures 2A, B**) except for a single highly susceptible sample. However, total plasma cell frequencies were significantly reduced at the 200ng dose of soluble receptor in mock cultures but were not affected by IL-21 neutralization in KSHV-infected cultures (**Figure 2C**). Other subsets that were influenced by IL-21 supplementation (**Figure 1G**) were not affected by neutralization using soluble IL-21 receptor (**Supplementary Figure 3C**). Thus, IL-21 neutralization decreases plasma cell frequencies in the absence of infection, but KSHV can compensate for this by independently supporting plasma cell frequencies, consistent with our results shown in **Figure 1G**. Thus, our data demonstrate that IL-21 neutralization generally has no effect on overall infection, but also does not inhibit KSHV-mediated support of plasma cell numbers. Taken together our supplementation and neutralization datasets support the conclusion that the effect of IL-21 on early KSHV infection is directly related to an IL-21 mediated increase in plasma cell frequencies.

### 3.4 IL-21 receptor expression on plasmablasts and naïve B cells correlates with increased KSHV infection

**Figure 3:**
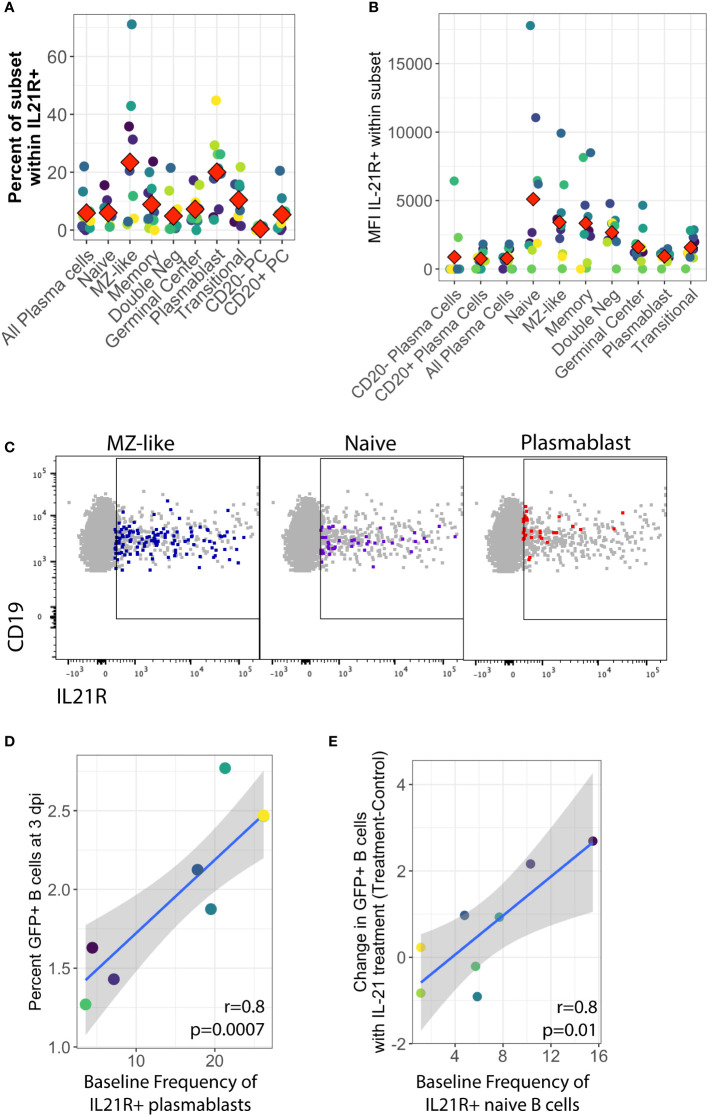
Baseline IL-21 receptor distribution in primary human tonsil B lymphocytes and its correlation to KSHV susceptibility and the response to IL-21 supplementation. B cell immunophenotyping analysis including IL-21R was performed at baseline (Day 0) for 10 unique tonsil specimens (**Supplementary Figure 1B**). **(A)** Percent of individual B cell subsets within viable/CD19+/IL-21+. Red diamonds indicate the mean value for all tonsil specimens **(B)** MFI of IL-21 receptor within B cell subsets. Red diamonds indicate the mean values for each subset and colors indicate specific tonsil specimens and can be compared between panels **(A, B, D, E)**. **(C)** Representative flow cytometry data showing viable/CD19+ B cells (grey) and IL-21R+ subsets as indicated by panel label (colored overlay) **(D)** Pearson correlation analysis of baseline IL-21R+ plasmablasts with total GFP+ B cells at 3 dpi. **(E)** Pearson correlation analysis of baseline IL-21R+ naïve B cells with the effect of IL-21 supplementation on overall KSHV infection in the same tonsil specimens at 3dpi. Grey shading indicates 95% confidence interval.

To further address the early stages of the IL-21 response during infection, we examined expression of the IL-21 receptor (IL-21R) in primary human tonsil B lymphocytes at baseline (day 0) in each tonsil specimen. We observed that IL-21 receptor expression is rare on B cells in tonsil, averaging less than 3% of total viable B cells in most samples.The distribution of IL-21 receptor positive cells among B cell sub-populations is broad and varies substantially between samples, but IL-21 receptor-expressing B cells are most likely to have an MZ-like or plasmablast immunophenotype (**Figures 3A, C**) and on a per-sample basis, either plasmablast or MZ-like subsets dominated the IL-21R positive cells in most tonsil samples (**Supplementary Figure 3D**). When we examined subset-specific levels of IL-21R *via* the geometric mean fluorescence intensity of the IL-21R staining within IL-21R+ cells in each subset, we observed that naïve, MZ-like and memory subsets (classical memory and double negative) expressed the highest levels of IL-21R and thus may be most responsive to IL-21 signaling (**Figures 3B, C**).

In order to determine whether IL-21 receptor expression prior to infection influenced the establishment of KSHV infection, we aggregated the untreated conditions from both the supplementation and the neutralization experiments and examined correlations between baseline IL-21R distribution and KSHV infection based on overall GFP. This analysis revealed that the proportion of plasmablasts within IL-21R+ B cells is significantly correlated with overall susceptibility to KSHV infection in the absence of any IL-21 treatment (r=0.81, p=0.0007) (**Figure 3D**). However, in experiments where we supplemented cultures with IL-21, the IL-21-mediated increase in overall KSHV infection at 3 dpi (**Figure 1C**) is correlated with the baseline frequency of IL-21 receptor expression on naïve B cells (r=0.82, p=0.01) (**Figure 3E**). These results suggest that IL-21+ plasmablasts are important for susceptibility to KSHV in the absence of high levels of IL-21 at early timepoints in untreated cultures while naïve B cells contribute to the effect of IL-21 supplementation.

Interestingly, there was no positive correlation seen between baseline IL-21R expression and the response of plasma cell frequencies to IL-21, suggesting that the plasma cell response to IL-21 at 3 dpi in KSHV infected cultures may be a product of IL-21 receptor up-regulation in response to infection instead of intrinsic baseline levels of IL-21R on plasma cells in our tonsil lymphocyte cultures. Indeed, modulation of IL-21 receptor expression by KSHV infection is one possible mechanism for the synergistic promotion of plasma cell numbers we observe with both IL-21 treatment and infection (**Figure 1E**).

### 3.5 IL-21 receptor expression on plasmablasts and plasma cells is altered by KSHV infection.

**Figure 4:**
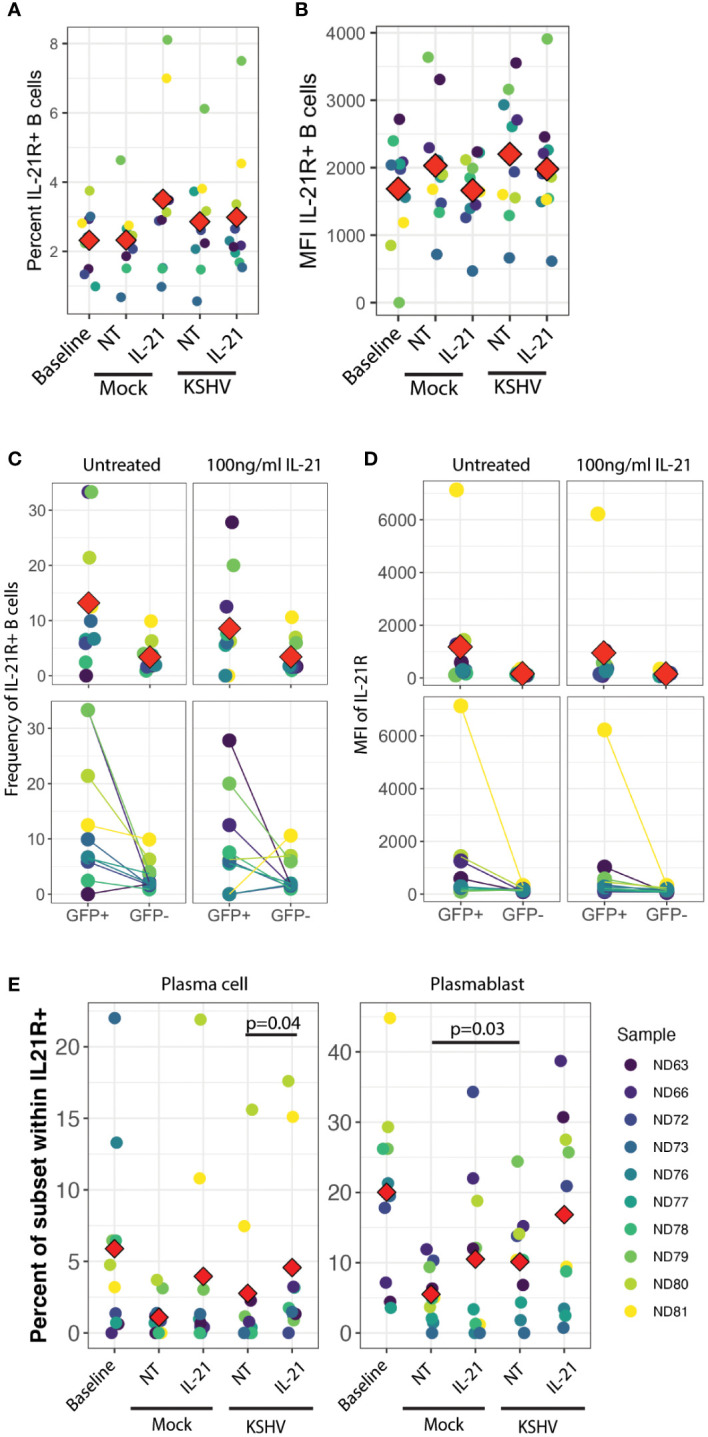
IL-21R+ plasmablasts increase in response to KSHV infection and IL-21R+ plasma cells increase in response to IL-21 only in KSHV+ cultures. **(A)** Total percentage of viable/CD19+/IL-21R+ B cells at baseline and 3dpi within Mock, Mock+100pg/ml IL-21, KSHV, KSHV+ 100pg/ml IL-21 conditions. **(B)** conditions as in **(A)** for mean fluorescence intensity of IL-21R staining in IL-21R+ B cells **(C)** Frequency and **(D)** MFI of IL-21R for IL-21R+ B cells within viable/CD19+/GFP+ or viable/CD19+/GFP- in untreated or 100pg/ml IL-21-treated cultures at 3 dpi Top panels in **(C)** and **(D)** show individual sample quantities and means (red diamonds) and bottom panels show trends of increase/decrease comparing GFP+ to GFP- within the same culture. **(E)** Distribution of plasma cell (left) or plasmablast (right) within viable/CD19+/IL-21R+ at day 0 (baseline) or 3dpi within Mock, Mock+100pg/ml IL-21, KSHV or KSHV+ 100pg/ml IL-21 conditions. Red diamonds indicate the mean values for each condition and significant differences were assessed via two-way repeated measures ANOVA and *post-hoc* paired T-tests for both culture/infection conditions and IL-21 treatment (shown). For all panels Red diamonds indicate group means and colors indicate individual tonsil specimens and can be compared between all panels in the figure.

In order to examine this hypothesis, we analyzed IL-21R expression on B cell subsets at 3 dpi in our culture system with or without IL-21 supplementation to determine whether KSHV and/or IL-21 can modulate the response to IL-21 during infection by modifying expression of IL-21R. There were no statistically significant differences in either the frequency (**Figure 4A**) or fluorescence intensity (**Figure 4B**) of IL-21R with KSHV infection or IL-21 supplementation. When we examined IL-21R expression on KSHV-infected (GFP+) cells vs GFP- cells in the same culture, we observed non-significant trends in the data showing that GFP+ cells were more likely to be IL-21R+ (**Figure 4C**) and had higher MFI for IL-21R expression (**Figure 4D**) compared to GFP- cells in the same culture. Neither of these effects was altered by IL-21 stimulation. When we examined the distribution of B cell subsets within IL-21R+ B cells, the majority of effects on IL-21R expression at 3 dpi were present in both Mock and KSHV-infected cultures, indicating they are a product of the culture system and not driven by KSHV. However, the proportion of plasmablasts within IL-21R+ cells was significantly increased comparing Mock to KSHV-infected cultures without IL-21 treatment (p=0.03) and this difference was further increased with the combination of KSHV infection and IL-21 treatment (**Figure 4E**, right). Comparing untreated and IL-21 treated cultures within infection conditions revealed a significant effect of IL-21 treatment on IL-21+ plasma cells only in the KSHV-infected cultures (**Figure 4E**, left).

These results may indicate that infection and IL-21 treatment is affecting IL-21R expression on existing plasmablasts and plasma cells, or that KSHV and IL-21 synergistically drive differentiation of IL-21R+ cells to plasmablast and plasma cell phenotypes. Our observation that IL-21R+ naïve B cells at day 0 are correlated with the response of KSHV infection to IL-21 treatment (**Figure 3E**) is one indication that differentiation may be playing a role in the IL-21 response. However, our data do not exclude the possibility that a combination of both receptor modulation and differentiation are contributing to the observed 3 dpi phenotypes in the presence of both IL-21 and KSHV infection.

### 3.6 IL-21 and KSHV promote B cell proliferation and plasma cell differentiation

**Figure 5:**
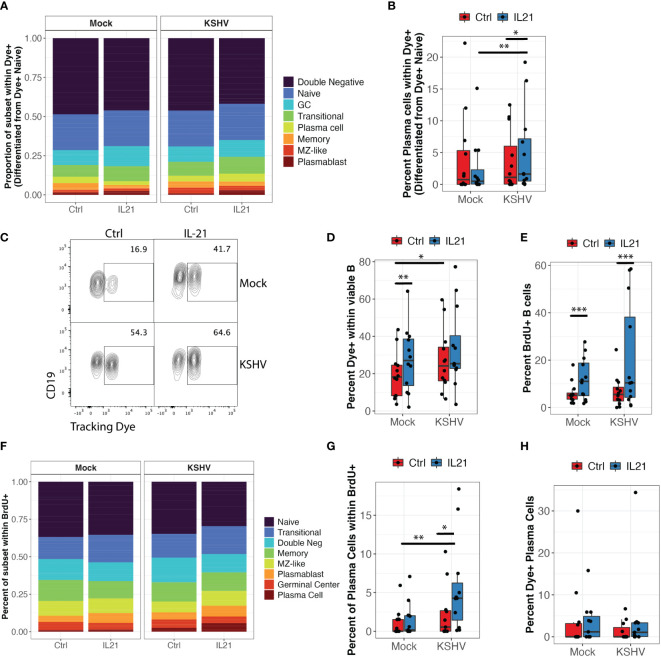
KSHV and IL-21 promote B cell proliferation and differentiation of naïve B cells into plasma cells. Naïve B cells from 12 unique tonsil donors were isolated, loaded with a tracking dye, mock infected or infected with KSHV and re-constituted into the total lymphocyte environment with or without IL-21. At 3dpi, B cell immunophenotyping was performed to identify the differentiated phenotype of dye+ cells. **(A)** proportional immunophenoptypes of B cells that differentiated from dye+ naïve precursors **(B)** Data as in **(A)** showing the frequency of newly-differentiated dye+/CD138+ plasma cells. Two-way repeated measures ANOVA shows a significant interaction effect of KSHV and IL-21 treatment on differentiation of naïve B cells into CD138+ plasma cells (p=0.03, F=5.9). *Post-hoc* Wilcoxon rank sum test shows *p=0.007 comparing Mock to KSHV within IL-21 treated conditions and *p=0.03 comparing untreated and IL-21 treated within KSHV infected cultures. **(C)** Representative flow cytometry plot showing the effect of KSHV infection and IL-21 treatment on total dye+ cells at 3 dpi **(D)** summary data for 12 tonsil specimens showing total viable/CD19+/dye+ cells at 3dpi. Two-way repeated measures ANOVA shows significant affects of both infection (p=0.03, F=5.9) and IL-21 treatment (p=0.007, F=11.1). *Post-hoc* Wilcoxon rank sum test shows **p=0.001 comparing untreated and IL-21 treated within Mock cultures and *p=0.02 comparing Mock and KSHV infected within untreated cultures. **(E)** Total frequency of viable/CD19+/BrdU+ cells under the indicated conditions. Two-way repeated measures ANOVA shows a significant effect of IL-21 treatment in both Mock and KSHV-infected conditions (p=0.02, F=7.0). *Post-hoc* Wilcoxon rank sum test shows ***p=0.001 comparing treated to untreated in both Mock and KSHV infected cultures. **(F)** Proportional distribution of B cell subsets within viable/CD19+/BrdU+ populations at 3dpi. **(G)** Two-way repeated measures ANOVA shows a significant effect of IL-21 (p=0.003, F=15.4) and a significant interaction of KSHV and IL-21 (p0.02, F=8) on BrdU+ plasma cells. *Post-hoc* Wilcoxon rank sum test shows *p=0.03 comparing treated to untreated in KSHV cultures and **p=0.003 comparing Mock to KSHV within IL-21 treated cultures. Similar experiments were performed in 12 tonsil samples where CD138+ plasma cells were isolated and loaded with tracking dye prior to infection and treatment. **(H)** Percent of viable/CD19+/CD138+ plasma cells at 3dpi that contained the tracking dye (surviving mature plasma cells).

We next wanted to characterize the mechanisms of increased plasma cell frequencies in our KSHV-infected, IL-21 treated cultures (**Figure 1E**) which correlates strongly with the positive effect of IL-21 on overall KSHV infection at 3 dpi (**Figure 1H**, left). We hypothesized that IL-21 is driving differentiation of new plasma cells over time, and that KSHV infection would increase this differentiation. However, immunophenotyping by flow cytometry provides a snapshot of the subset defining markers on any individual B cell at the analysis timepoint, but does not allow us to know what the immunophenotype of that B cell was at any prior timepoint. Thus, to test our hypothesis that differentiation is playing a role in the IL-21 phenotype we employed a tracking dye to label only naïve B cells at the start of the experiment. In this way, dye positive cells observed at 3 dpi that have immunophenotypic markers consistent with more mature subsets (e.g. CD27, CD38, CD138) must be naïve B cells that have differentiated to acquire these mature immunophenotypes over the experimental timecourse. Results from naïve B cell tracking experiments with 11 unique tonsil specimens show that differentiation of naïve B cells does occur in our *ex vivo* culture system. Most naïve B cells acquired an atypical double negative memory immunophenotype or remained phenotypically naïve over the experimental timecourse. However, we did observe dye+ cells for other lineages (**Figure 5A**). Importantly, most of the differentiation effects were similar between Mock and KSHV-infected cultures and between IL-21 treated and un-treated cultures. However, a two-way repeated measures ANOVA revealed a significant interaction of KSHV infection and IL-21 treatment (p=0.03, F=5.9) on the differentiation of naïve B cells into CD138+ plasma cells in these experiments (**Figure 5B**).

Interestingly, ANOVA analysis also revealed significant main effects of infection (p=0.03, F=5.9) and IL-21 treatment (p=0.007, F=11.1) on the total frequency of dye positive cells at 3dpi but there was no significant interaction (**Figures 5C, D**). In order to determine whether the increase in total dye+ cells was indicative of proliferation or survival, we analyzed BrdU incorporation in these cultures. IL-21 treatment significantly increased BrdU positive B cells in both Mock and KSHV-infected conditions (**Figure 5E**; p=0.02, F=7.0 by two-way repeated measures ANOVA). Immunophenotyping of BrdU+ cells indicates that all lineages show a proliferation history to some extent, but naïve and transitional B cells are the most proliferative in our tonsil cultures (**Figure 5F**). When we performed a two-way repeated measures ANOVA to determine whether IL-21 or KSHV infection was significantly affecting the proliferation of any B cell subsets, we observed a significant main effect of IL-21 treatment on BrdU+ plasma cells (p=0.003, F=15.4) as well as a significant interaction of IL-21 and KSHV infection on BrdU+ plasma cells (p=0.02, F=8) (**Figure 5G**). These results demonstrate that naïve B cells are both proliferating and differentiating in response to KSHV infection and IL-21 stimulation. In order to determine the contribution of pre-existing plasma cells to the IL-21 effect, when we performed similar experiments where CD138+ plasma cells were loaded with tracking dye to monitor their survival and response to infection and IL-21 stimulation. Importantly, the percentage of CD138+ cells in these cultures that were dye+ at 3dpi was generally low (>5%) and not significantly affected by IL-21 treatment or KSHV infection (**Figure 5H**), indicating that the majority of plasma cells we observe at 3 dpi are newly-differentiated. Taken together, these experiments demonstrate that IL-21 and KSHV infection synergistically drive proliferation and differentiation of naïve B cells into plasma cells. Moreover, the majority of the plasma cells present at 3dpi are newly-differentiated, thus supporting the conclusion that differentiation is the primary mechanism driving the synergistic increase in plasma cell frequencies we observed in our IL-21 supplementation experiments (**Figure 1E**).

### 3.7 IL-21 production by CD8 central memory T cells correlates with plasma cell frequencies and targeting

**Figure 6:**
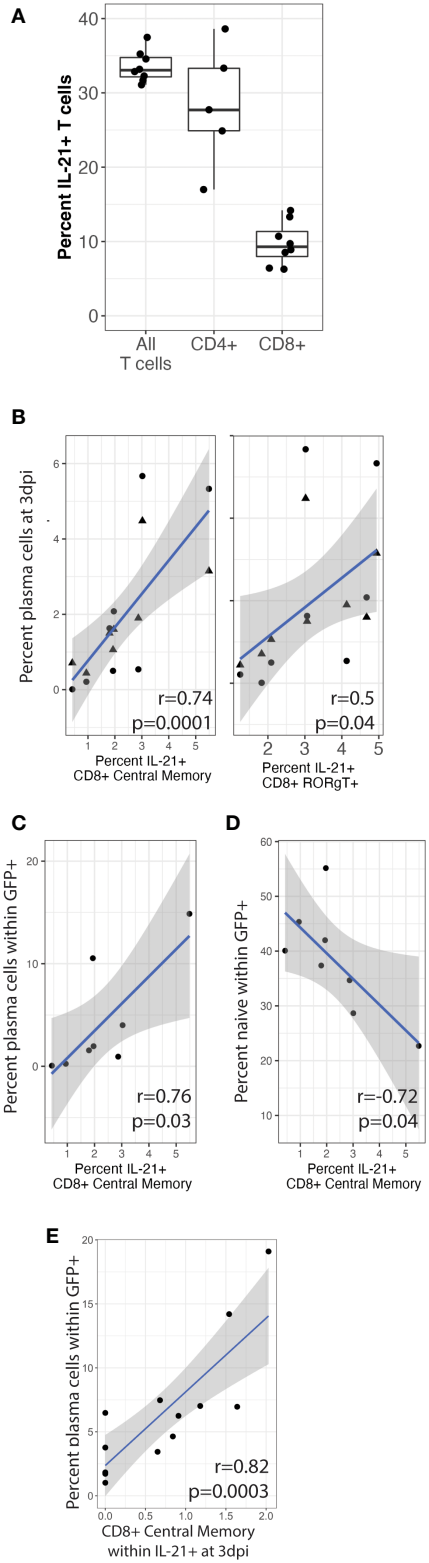
CD8+ central memory T cells producing IL-21 correlate with plasma cell frequencies and targeting. T cells were analyzed by surface immunophenotyping, intracellular transcription factor staining and ICCS for IL-21 secretion (**Supplementary Figure 2**) in Mock and KSHV-infected total lymphocyte cultures at baseline (day 0) in 8 unique tonsil specimens. **(A)** Frequency of total, CD4+ and CD8+ IL-21+ T cells **(B)** Percent of IL-21+ CD8+ central memory (CD8-CM) and CD8+ RORgT+ T cells correlated with CD138+ plasma cells at 3dpi in the same tonsil specimen in mock (triangles) or KSHV infected (circles) cultures. IL-21+ CD8-CM correlated with **(C)** the proportion of plasma cells and **(D)** the proportion of naïve B cells within the infected population at 3 dpi. T cells were analyzed as above for immunophenotypes and IL-21 secretion at 3dpi for 12 unique tonsil specimens. **(E)** IL-21+ CD8-CM frequencies correlated with infection in plasma cells. Pearson correlation coefficients and p-values are shown on the figure panels, blue lines are linear model regressions and grey shading is 95% confidence intervals.

We next wanted to determine the sources of native IL-21 secretion in our culture system and determine whether IL-21 producing T cell subsets are correlated with plasma cell frequencies or targeting at 3 dpi, which would indicate that native IL-21 can influence KSHV infection even though such effects were too subtle to observe experimentally using IL-21 neutralization in our experiments (**Figure 2**). To accomplish this, we utilized an additional immunophenotyping panel for T cell subsets (**Table 1** and **Supplementary Figure 2A**) and performed intracellular cytokine staining (ICCS) on unstimulated T cells at day 0 from 8 tonsil donors to identify T cell subsets that are producing IL-21 (**Supplementary Figure 2B**). Intracellular IL-21 was observed in 30-40% of T cells and a greater proportion of CD4+ T cells were expressing IL-21 compared to CD8+ (**Figure 6A**). When we analyzed specific T cell subsets expressing IL-21 and looked for correlations to total plasma cell numbers at 3 dpi, we found that CD8+ central memory (r=0.74, p=0.001; σ=0.82, p=0.0001) and CD8+ RORgT+ (r=0.5, p=0.04; σ=0.69, p=0.003) were significantly correlated (**Figure 6B**). Moreover, the baseline frequency of IL-21 positive CD8+ central memory T cells was also significantly correlated with KSHV infection of plasma cells at 3dpi (r=0.76, p=0.03; σ=0.76, p=0.04) and negatively correlated with infection of naïve B cells (r=-0.72, p=0.04) (**Figures 6C, D**). Consistent with these results, IL-21 secretion by CD8+ central memory T cells was also significantly correlated with infection of plasma cells (r=0.82, p=0.0003) in an independent dataset where we examined IL-21 secretion at 3 dpi in conjunction with KSHV infection (**Figure 6E**). These results demonstrate that a large proportion of T cells isolated from tonsil are producing IL-21 and there is significant correlation between specific IL-21 producing CD8+ T cell subsets and the phenotypes we observed in IL-21 supplementation experiments. Thus, it is possible that, in combination with other immunological factors, IL-21 may influence KSHV infection at physiological levels in samples where specific milieus including IL-21 producing T cells provide an advantageous environment for the establishment of infection.

## 4 Discussion

Dysregulation of the inflammatory environment is a hallmark of all KSHV-associated malignancies (29–31). However, the role of the baseline inflammatory environment in host-level susceptibility and the contribution to host cytokines to transmission and the establishment of infection have not been examined. In this study, we uncovered a role for IL-21 signaling in the establishment of KSHV infection in tonsil lymphocytes. The relationship between plasma cell differentiation and IL-21 is well-characterized in human immunology (14, 15), and we previously showed that plasma cells are highly targeted during early KSHV infection (10). However, the results presented in this study are the first direct evidence that B cell differentiation into plasma cells plays a role in early infection events for KSHV.

These results are consistent with previous findings in the murine gamma herpesvirus MHV-68 model where Collins and Speck recently used IL-21R knockout mice to demonstrate that IL-21 signaling is critical for the establishment of MHV68 latency specifically in B cells. This study showed that the mechanisms of decreased infection were related to decreases in both germinal center and plasma cell frequencies as well as decreased infection in both the germinal center and plasma cell compartment at later timepoints post-infection (20), suggesting a critical mechanism for IL-21 in MHV68 transit of the germinal center and differentiation of follicular-derived plasma cells.

Our results support a similar role for IL-21 signaling in the early stages of KSHV infection in tonsil. Interestingly, our results imply that the successful differentiation of plasma cells has an influence on the overall establishment of infection in all B cell subsets. This is similar to mechanisms previously described for EBV where naïve B cells are infected and transit the germinal center, thereafter acquiring a plasmablast, memory or plasma cell phenotype (32). For EBV, reaching the plasma cell stage is associated with lytic reactivation (33). In our bulk RT-PCR data, the link between plasma cell differentiation and increased overall infection could not be attributed to increased lytic gene expression. However, we previously showed *via* single cell RT-PCR that plasma cells display a mixture of lytic and latent phenotypes at 3 dpi (10), which would support a model where newly-differentiated plasma cells are producing KSHV which then targets other B cell subsets.

Readers should note that we have used a broad definition of “plasma cell” in this study; gating all CD19+ CD138+ cells as plasma cells. Canonically, human plasma cells should be defined based on the more stringent definition as CD19+, CD38+, CD27+, CD138+. However, we have chosen to capture all CD138+ cells in our analysis in order to allow for non-standard differentiation pathways induced by KSHV infection and/or our *ex vivo* culture system. Indeed, KSHV-associated lymphomas display a wide variety of characteristics and immunophenotypes *in vivo* and KSHV-PEL are commonly CD138+ with no other B cell-defining immunophenotypic markers. It has been theorized that this variety of phenotypes may be influenced by the presence or absence of co-pathogens (e.g. EBV and HIV) as well as the variable immunological states under which KSHV lymphoproliferations occur (34). Moreover, there is evidence of dysregulated GC reactions and non-standard extrafollicular differentiation pathways associated with KSHV and other gamma-herpesviruses, which could contribute to the acquisition of atypical immunophenotypes in these contexts [reviewed in (35)].

Our results herein implicate a particular T cell milieu in promoting plasma cell frequencies and plasma cell targeting during early KSHV infection. Our current analysis demonstrates IL-21+ CD8+ central memory T cells at both baseline and 3dpi are correlated to the frequency and infection of plasma cells. Given that CD8+ T cells represent a minority of the IL-21 producing cells in our cultures, this data is difficult to interpret. However, the correlation was reproducible in two independent datasets at both baseline and 3 dpi, increasing our confidence in this association. These results may imply that increased levels of IL-21 positive CD8+ central memory T cells are a hallmark of a particular inflammatory state that favors plasma cell differentiation and KSHV infection based on additional factors that are yet unidentified. Previous studies have shown that CD8+ T cells can be observed in B lymphocyte areas of tonsil and provide co-stimulatory signals and cytokines to support B cell survival (36). Interestingly, recent studies have shown that IL-6 regulates IL-21 production in CD8+ T cells in a STAT3-dependent manner, and that CD8+ T cells induced in this way can effectively provide help to B cells (37). Thus, the induction of human IL-6 during KSHV infection may modulate the function of CD8+ T cells in a way that favors the establishment and dissemination of KSHV infection within the lymphocyte compartment independent of traditional CD4+ helper T cells, which would be an interesting dynamic in the context of CD4+ T cell immunosuppression associated with HIV infection where KSHV-mediated malignancies are common.

Based upon our current data, we would hypothesize that the T cell composition of tonsil samples at baseline can be used to predict sample-level susceptibility to KSHV infection. Epidemiological evidence from Africa suggests that acquisition of KSHV infection in infants is lower than expected based on shedding of KSHV by household contacts, indicating that unknown factors influence the initial acquisition of KSHV infection in childhood (38). Our results at least implicate an immunologically activated state in the initial establishment of KSHV infection in tonsil lymphocytes, suggesting that prior pathogen exposure, chronic infection or temporally-associated acute infections may create an inflammatory state in the tonsil that is permissive for KSHV transmission.

## Data availability statement

The raw data supporting the conclusions of this article will be made available by the authors, without undue reservation.

## Ethics statement

Human specimens used in this research were de-identified prior to receipt, and thus were not subject to IRB review as human subject’s research.

## Author contributions

NA and JT contributed to the conception and design of the study. NA performed experiments and data analysis. JT contributed to analysis, statistical analysis, data visualization and funding for the study. NA wrote the first draft of the manuscript. All authors contributed to the article and approved the submitted version.

## Supplementary material

Supplementary Figure 1 B cell gating scheme and controls.

Supplementary Figure 2 T cell gating scheme and controls.

Supplementary Figure 3 Supplementary data panels.

Supplementary Table 1 Count data from flow cytometry for the IL-21 supplementation dataset (**Figure 1**).
