# Supplementary figures and images for: IL-21 signaling promotes the establishment of KSHV infection in human tonsil lymphocytes by increasing differentiation and targeting of plasma cells
Source: Front Immunol. 2022 Dec 7;13:1010274. doi: 10.3389/fimmu.2022.1010274 (PMC9769966; doi:10.3389/fimmu.2022.1010274)

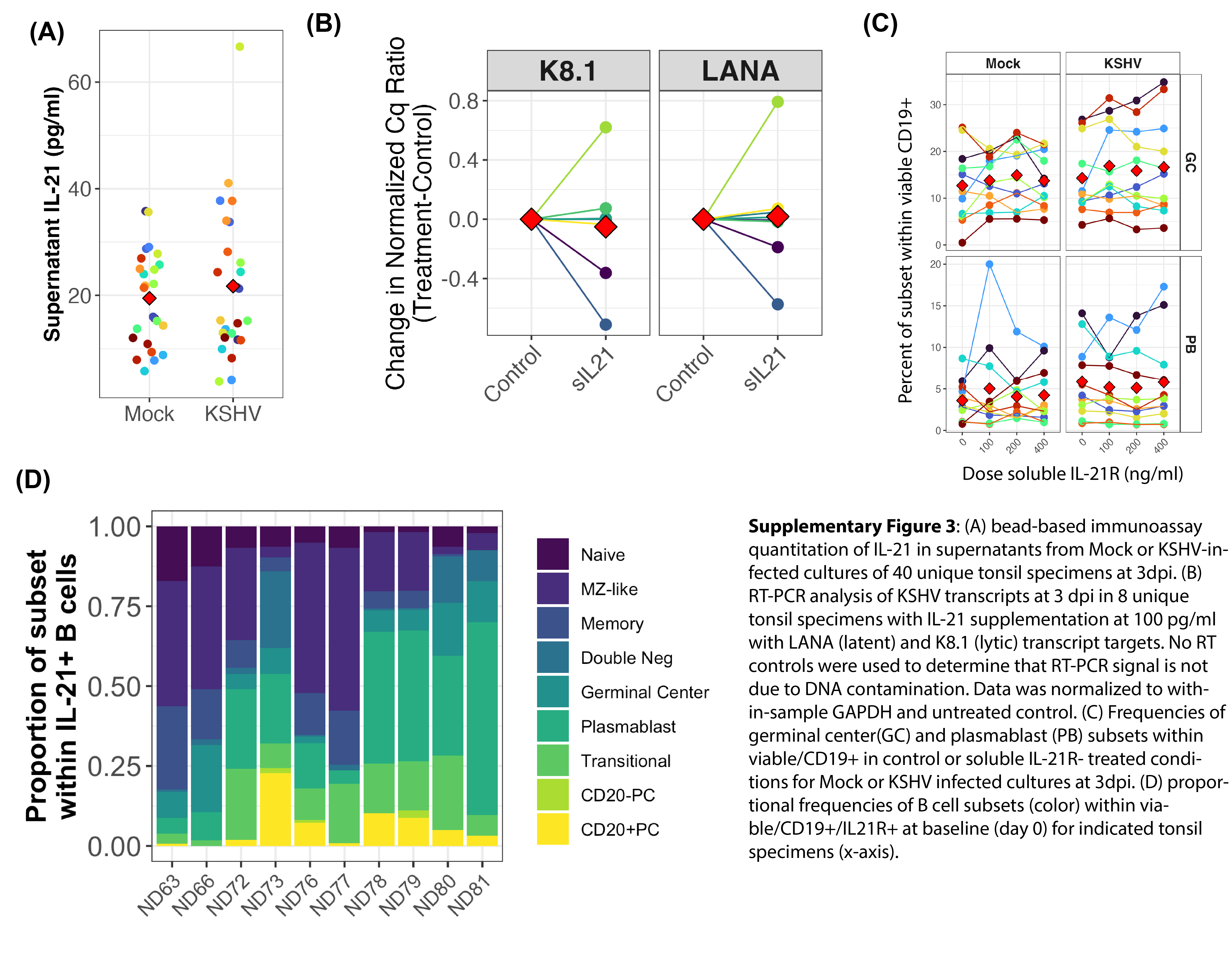

Supplement: Supplementary Figure 1 — B cell gating scheme and controls. [file Image_1.png]

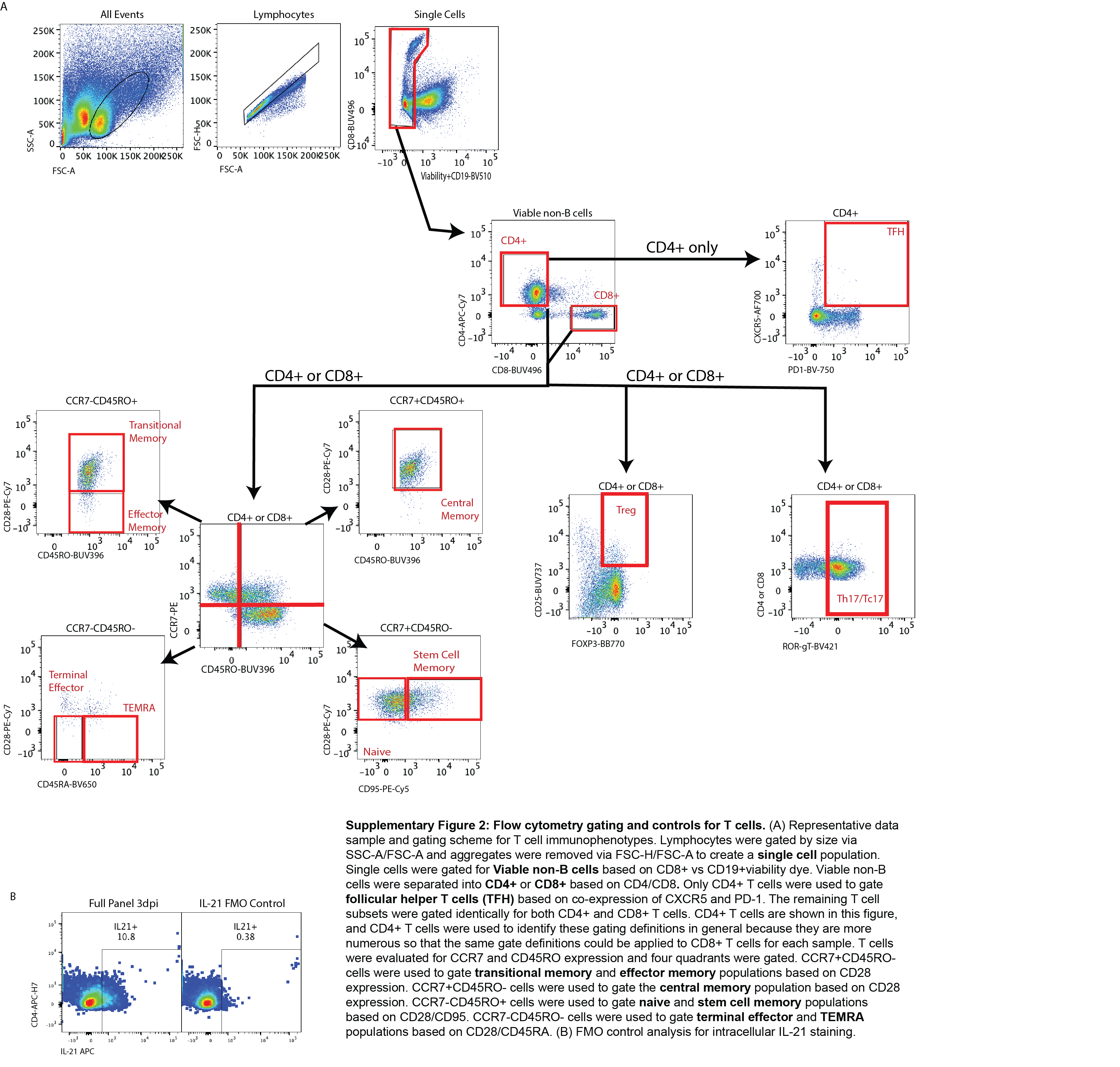

Supplement: Supplementary Figure 2 — T cell gating scheme and controls. [file Image_2.png]

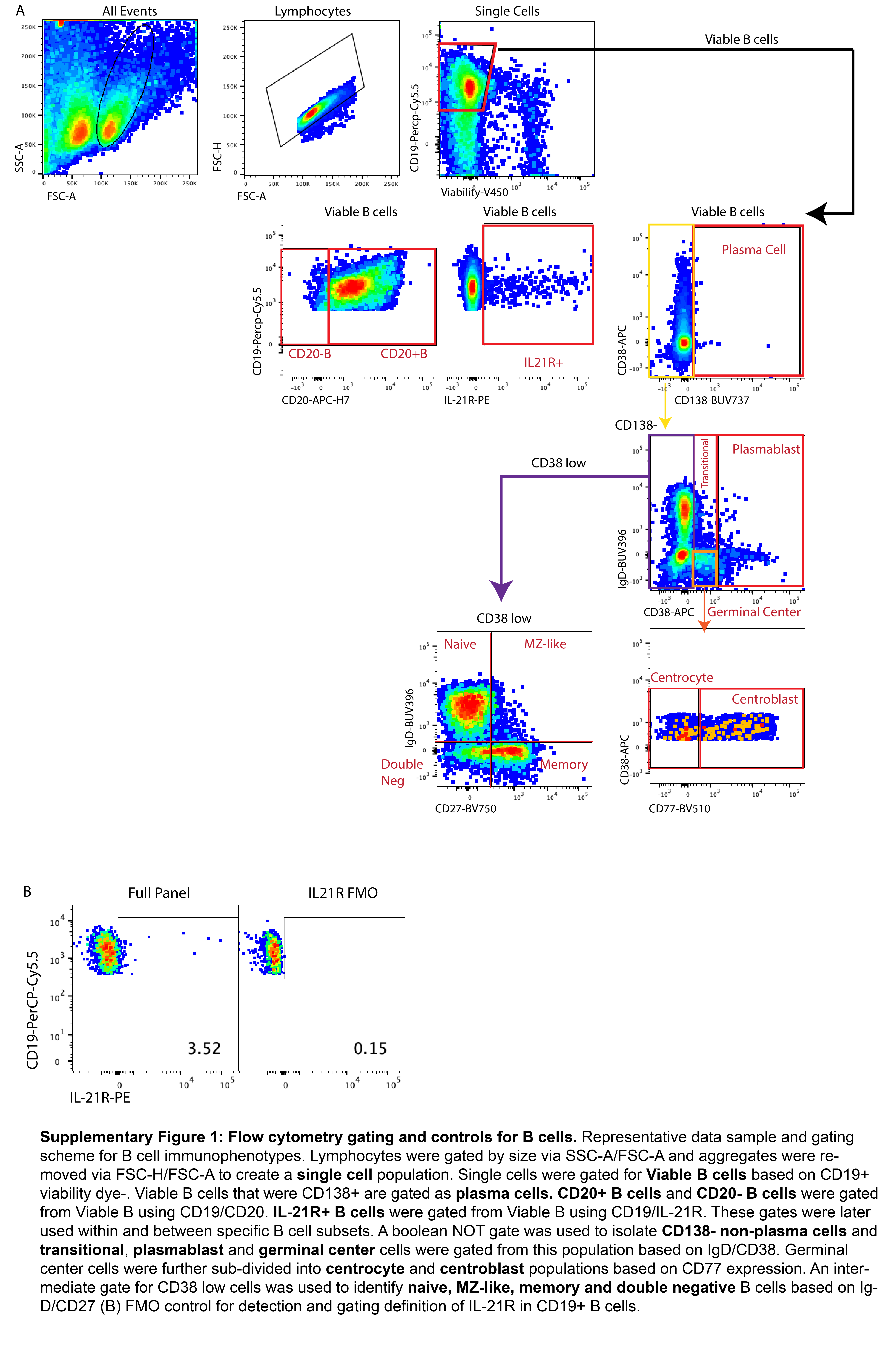

Supplement: Supplementary Figure 3 — Supplementary data panels. [file Image_3.png]
